# Studying the concentration of polymers in blended microplastics using 2D and 3D Raman mapping

**DOI:** 10.1038/s41598-023-35010-0

**Published:** 2023-05-12

**Authors:** Mehrdad Lotfi Choobbari, Jennifer Ferguson, Niko Van den Brande, Tim Smith, Tatevik Chalyan, Wendy Meulebroeck, Heidi Ottevaere

**Affiliations:** 1 Department of Applied Physics and Photonics, Brussels Photonics, Vrije Universiteit Brussel, Pleinlaan 2, 1050 Brussels, Belgium; 2 Renishaw plc, New Mills, Wotton-under-Edge, Gloucestershire, GL12 8JR UK; 3 Department of Materials and Chemistry, Physical Chemistry and Polymer Science, Vrije Universiteit Brussel, Pleinlaan 2, 1050 Brussels, Belgium; 4 Department of Applied Physics and Photonics, Brussels Photonics, Vrije Universiteit Brussel and Flanders Make, Pleinlaan 2, 1050 Brussels, Belgium

**Keywords:** Analytical chemistry, Optics and photonics

## Abstract

The combination of different polymers in the form of blended plastics has been used in the plastic industry for a long time. Nevertheless, analyses of microplastics (MPs) have been mainly limited to the study of particles made of single-type polymers. Accordingly, two members of the Polyolefins (POs) family, i.e., Polypropylene (PP) and Low-density Polyethylene (LDPE) are blended and extensively studied in this work due to their applications in industry as well as abundance in the environment. It is shown that 2-D Raman mapping only provides information about the surface of blended MPs (B-MPs). While complimentary 3-D volume analysis is needed to fully understand the presence of various polymers in such complex samples. Therefore, 3-D Raman mapping is applied to visualize the morphology of the distribution of polymers within the B-MPs together with the quantitative estimation of their concentrations. A parameter defined as the concentration estimate error (CEE) evaluates the precision of the quantitative analysis. Furthermore, the impact of four excitation wavelengths 405, 532, 633, and 785 nm is investigated on the obtained results. Finally, the application of a line-shaped laser beam profile (line-focus) is introduced for reducing the measurement time from 56 to 2 h.

## Introduction

Without any doubt, plastics are among the most useful materials for today’s industries. However, this economically friendly material has turned out to be a potential threat to our environment in past years^1^. A lot of attention has been paid to the investigation of plastic pollution, revealing striking data. It has been shown that a big island of plastic waste with an area of 1.6 million km^2^, almost three times the area of France, has been formed in subtropical waters between California and Hawaii over the past years^2^. Undoubtedly, with the current trend of consumption and the lack of proper methods for the disposal and recycling of plastic waste, the problem will further intensify in the coming years. For example, it has been estimated that 12,000 million metric tons of plastic waste will be present in the environment by 2050^3^. Besides that, reports are showing the presence of plastic particles, commonly referred to as microplastics (MPs), in the air we breathe^4^, in the food we eat^5^, in the water we drink^6^, and even in our body^7^. In fact, these tiny MPs (1 µm–5 mm) are mainly the result of the fragmentation of bigger plastic particles through different factors such as weathering, erosion, frictions, and ultraviolet (UV) light illumination, to name a few, that finally find their way to our daily life^8–10^.

Most of the studies dealing with the analysis of microplastics in the environment have mainly concentrated on the presence of single-type plastics such as Polyethylene (PE), Polypropylene (PP), Polystyrene (PS), etc.^11–13^. Whereas, tailoring the physicochemical characteristics of polymers for a specific application has been an established field of research for a long time^14^. Using this approach, polymers with desired features have been obtained without investing too much effort in inventing a totally new polymer, therefore, making it attractive for commercial purposes with a considerable market volume and production rate^15^. For instance, simple melt mixing of Polyolefins (POs), which constitutes a large family of commonly used polymers such as PP, PE, low-density PE (LDPE), and high-density PE (HDPE) has been a practical approach to improve the mechanical properties of final products without adding any compatibilizer^15,16^. The improved mechanical properties, however, may further elongate the required decomposition time of such plastic wastes which has been reported to be more than hundreds of years for single-type plastics^17^. Surprisingly, POs are among the most frequently detected types of MPs in the environment^5,12^. Having said that, only one group has recently investigated the presence of composite microplastics in the environment^18^. The authors applied 3-D Raman mapping on the composite microplastics composed of laminated layers of polymer and fiber to increase the reliability of identification. However, polymers can be used in the form of miscible and immiscible blends, possessing more complex compositions^19,20^. Reliable identification of different types of polymers that are present in such complex samples together with their quantitative analysis can be of great importance in the field, for example, for developing a standardized protocol for the analysis of microplastics^12,21^. Besides that, it has been reported that the study of blended plastics will increase the opportunity for their recycling, as various blends can form during the recycling process^22^.

The selection of a suitable analytical technique is a critical step for the study of blended microplastics (B-MPs). Apart from thermoanalytical methods that are capable of identifying the types of MPs^23^, Fourier-transform infrared spectroscopy (FTIR)^24^ and Raman spectroscopy (RS)^25^ seem to be more practical for the identification of complex B-MPs. FTIR, however, provides less resolution in comparison to the RS (10–20 µm against 1 µm), thus making this technique less attractive for this purpose^26,27^. It was recently shown that the resolution of FTIR can improve the analysis of MPs in the nanoscale regime when it is coupled with Atomic Force Microscopy (AFM)^28^. Nevertheless, this approach significantly increases the complexity and the cost of the analytical instrument, and it only provides 2-D information. Regarding thermoanalytical techniques, the plastic particles need to be destroyed during the measurement process, hence no information is gained about the morphology of polymers with these methods. Accordingly, we have adopted 2-D and 3-D Raman microspectroscopy (RMS) to analyze the B-MPs made from the POs family in this work. Concentration estimate analysis has been used to quantitatively evaluate the presence of each polymer within B-MPs in addition to visualizing the morphology of the distribution of polymers. Considering the dependence of the resolution of the Raman mapping on the applied laser wavelength, the results obtained with four different wavelengths have been compared qualitatively and quantitatively. Furthermore, the application of laser line-focus has been studied with the goal to shorten the required time of Raman mapping for the analysis of B-MPs.

## Results and discussion

### 2-D Raman mapping of B-MPs

**Figure 1:**
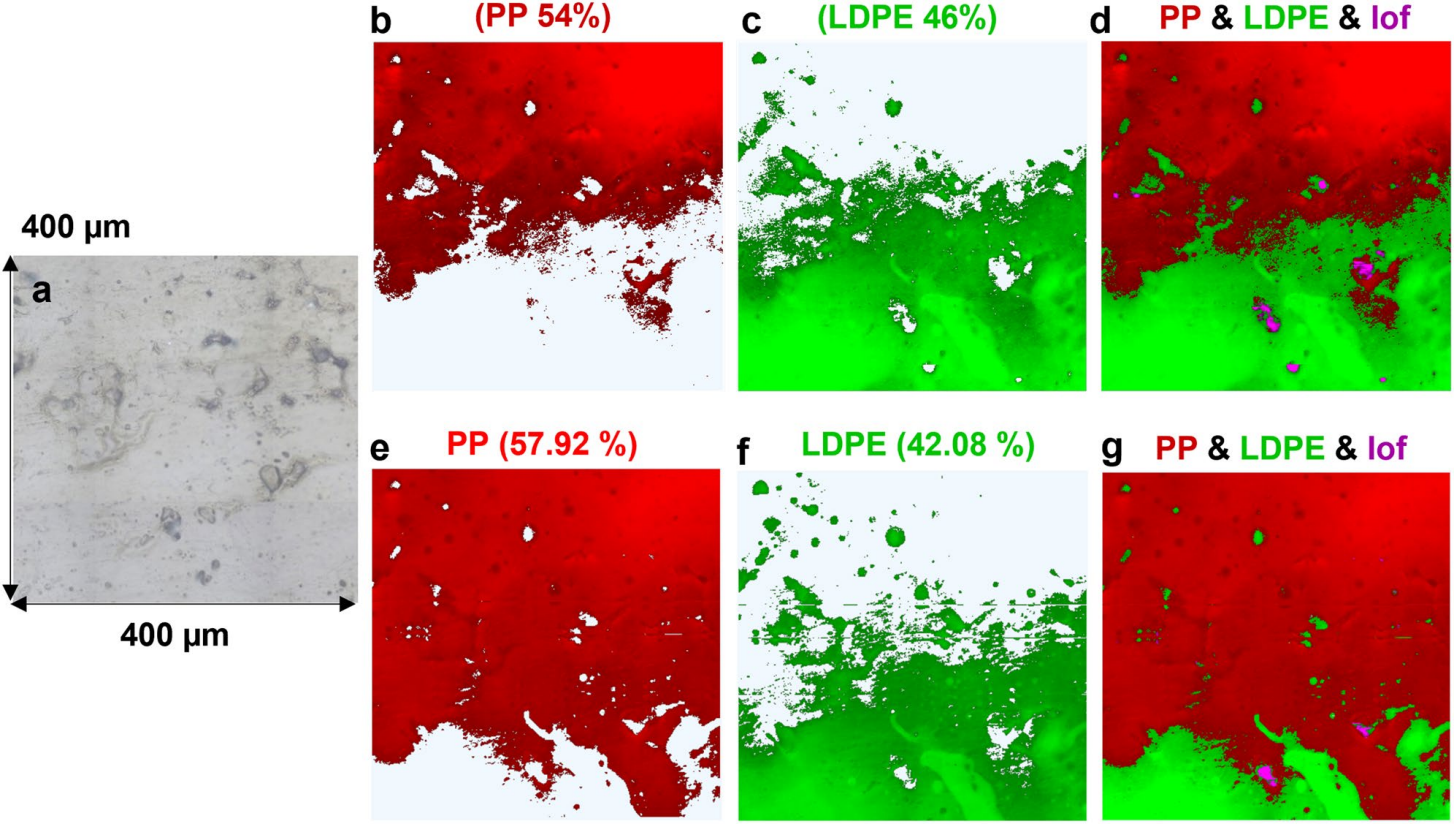
2-D Raman maps of PP/LDPE (75/25) obtained with 785 nm (top row) and 532 nm (bottom row) excitation wavelengths. (**a**) Shows the camera image of the mapped area, (**b**) and (**e**) show the distribution of PP together with its concentration in the corresponding area, (**c**) and (**f**) show the distribution of LDPE together with its concentration in the corresponding area, (**d**) and (**g**) show the combined maps of PP and LDPE together with lack of fit (lof) shown in purple color.

PP and LDPE blends have been widely used in different industrial applications such as packaging, fabrication of nets and ropes, as well as engineering polymers^29^. Accordingly, 2-D RMS was used to study the surface morphology of the polymers within PP/LDPE (weight ratios of 25/75 and 75/25) B-MPs together with a quantitative analysis of the concentration of each component. A certain area of each sample was 2-D Raman mapped with two laser wavelengths being 785 and 532 nm, keeping other measurement parameters consistent. Figure 1 demonstrates the 2-D Raman maps of PP/LDPE (75/25) that were obtained using 785 nm (top row) and 532 nm (bottom row) excitation wavelengths. Comparing the results reveals that Raman mapping with both excitation wavelengths yields information about the distribution of polymers within the analyzed B-MP. Nevertheless, the mapping performed with the 532 nm wavelength delivers a higher resolution Raman image, as expected^30^. For instance, a larger distribution of PP, that is elongated from the top of the mapped area to the bottom, is only recognized with the 532 nm wavelength when comparing the features in Fig. 1e,b. In addition, more small features of LDPE are visible on the top-left area of Fig. 1f compared to Fig. 1c. In fact, the 532 nm excitation wavelength gives a smaller focal point and a lower penetration depth compared to 785 nm excitation wavelength^18,30^. Thus, it provides a better resolution and excites a shallower surface. Consequently, the distribution of thin layers of polymers is more distinguishable using the 532 nm excitation wavelength. The results of the concentration estimate analysis are shown on top of each small image in Fig. 1. As seen, the amounts obtained using the 532 nm wavelength are closer to the known concentrations of PP and LDPE (75/25) in this B-MP than those obtained using the 785 nm wavelength. Nevertheless, the predicted concentrations in both scenarios are still far from the real amounts of each component within the analyzed B-MP, which could be due to two main reasons. First, the selected area for Raman mapping is not big enough and therefore, is not a good representative of the distribution of polymers. Second, the distribution of polymers on a 2-D surface may not be a good representative of their distribution within the volume of a B-MP. Indeed, the concentration gradient can happen during the melting and crystallization process of PP and LDPE^29^.

**Figure 2:**
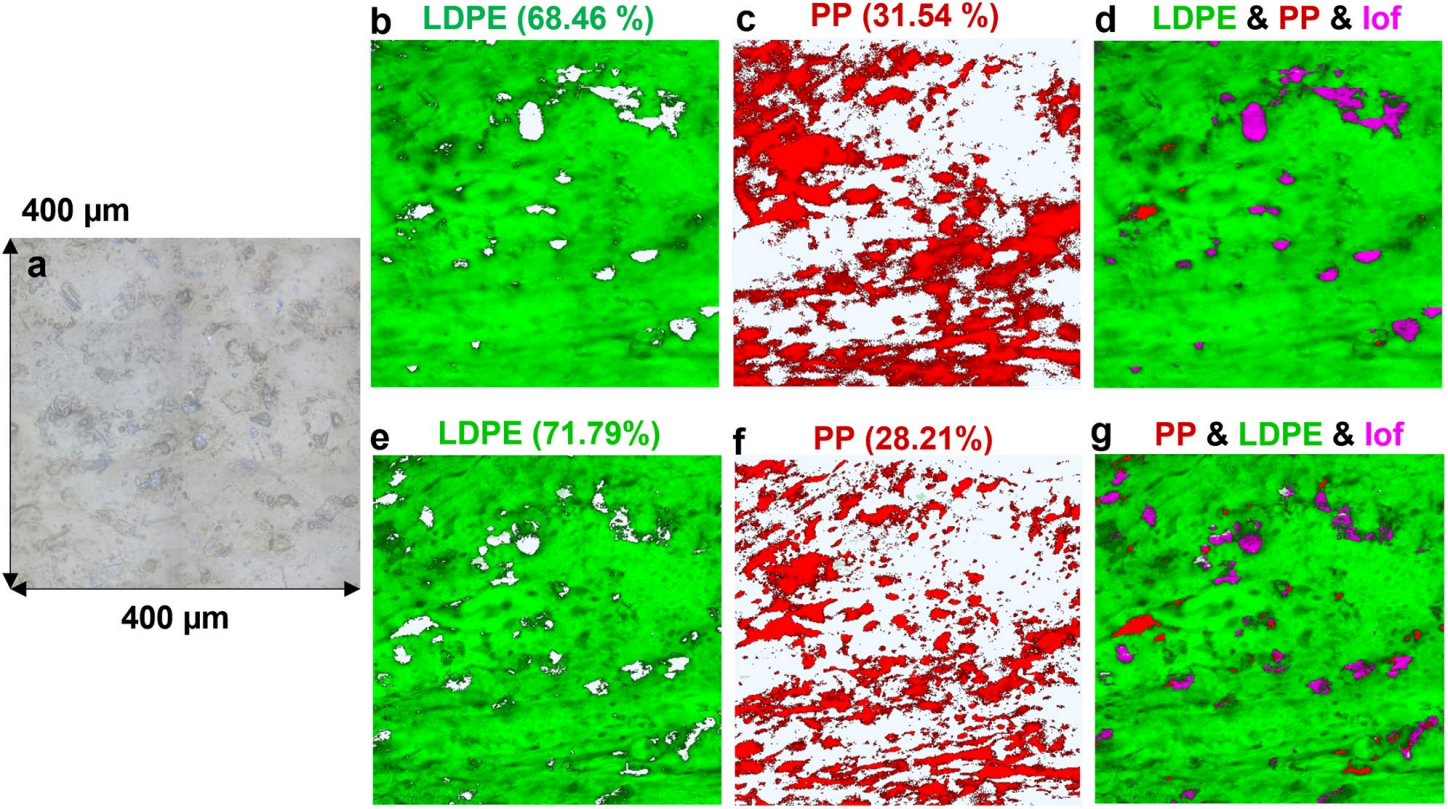
2-D Raman maps of PP/LDPE (25/75) obtained with 785 nm (top row) and 532 nm (bottom row) excitation wavelengths. (**a**) Shows the camera image of the mapped area, (**b**) and (**e**) show the distribution of LDPE together with its concentration in the corresponding area, (**c**) and (**f**) show the distribution of PP together with its concentration in the corresponding area, (**d**) and (**g**) show the combined maps of PP and LDPE together with lack of fit (lof) shown in purple color.

In the next step, the same analysis was performed on PP/LDPE (25/75) B-MP, the results of which are shown in Fig. 2. Likewise, the Raman maps obtained with 532 nm wavelength provided a better resolution for visualizing the distribution of polymers. This is more evident by comparing the sharpness of the features and extra artifacts in Fig. 2c,f. Moreover, the results of the concentration estimate analysis revealed again that a more precise evaluation of the concentration of polymers was obtained using 532 nm compared to the 785 nm excitation wavelength. This time, the predicted concentrations of PP and LDPE were closer to the real amounts of each component, i.e., 25% of PP and 75% of LDPE, within the analyzed B-MP. To validate these results, 2-D Raman mapping was performed on the other areas of both samples (see Fig. S1) and the Concentration Estimate Error (CEE) that is defined using the following formula was calculated.

$$CEE= \frac{\left|\left({RC}_{PP\,or\,LDPE}-{EC}_{PP\,or\,LDPE}\right)\right|}{{RC}_{PP\,or\,LDPE}}\times 100$$

where the *RC*_*PP or LDPE*_ and *EC*_*PP or LDPE*_ are the real (known) and the estimated concentrations of each polymer within the 2-D Raman maps, respectively. In total, the average CEEs for predicting the concentration of PP and LDPE polymers were found to be 25.86% and 42.70%, respectively, using the 2-D Raman mapping. Considering these results, one can draw a logical conclusion that additional complimentary information is required to fully understand the distribution of the polymers within the B-MPs. Even by mapping a very large area, still, a reliable estimation of the concentration of each constituent polymer may not be feasible within a B-MP. Besides that, a 2-D Raman map is not a good representative of the morphology of the constituent polymers throughout the different depths of a B-MP. Therefore, 3-D Raman mapping was applied for a more reliable analysis of B-MPs which is shown in the next session.

### 3-D Raman mapping of B-MPs

**Figure 3:**
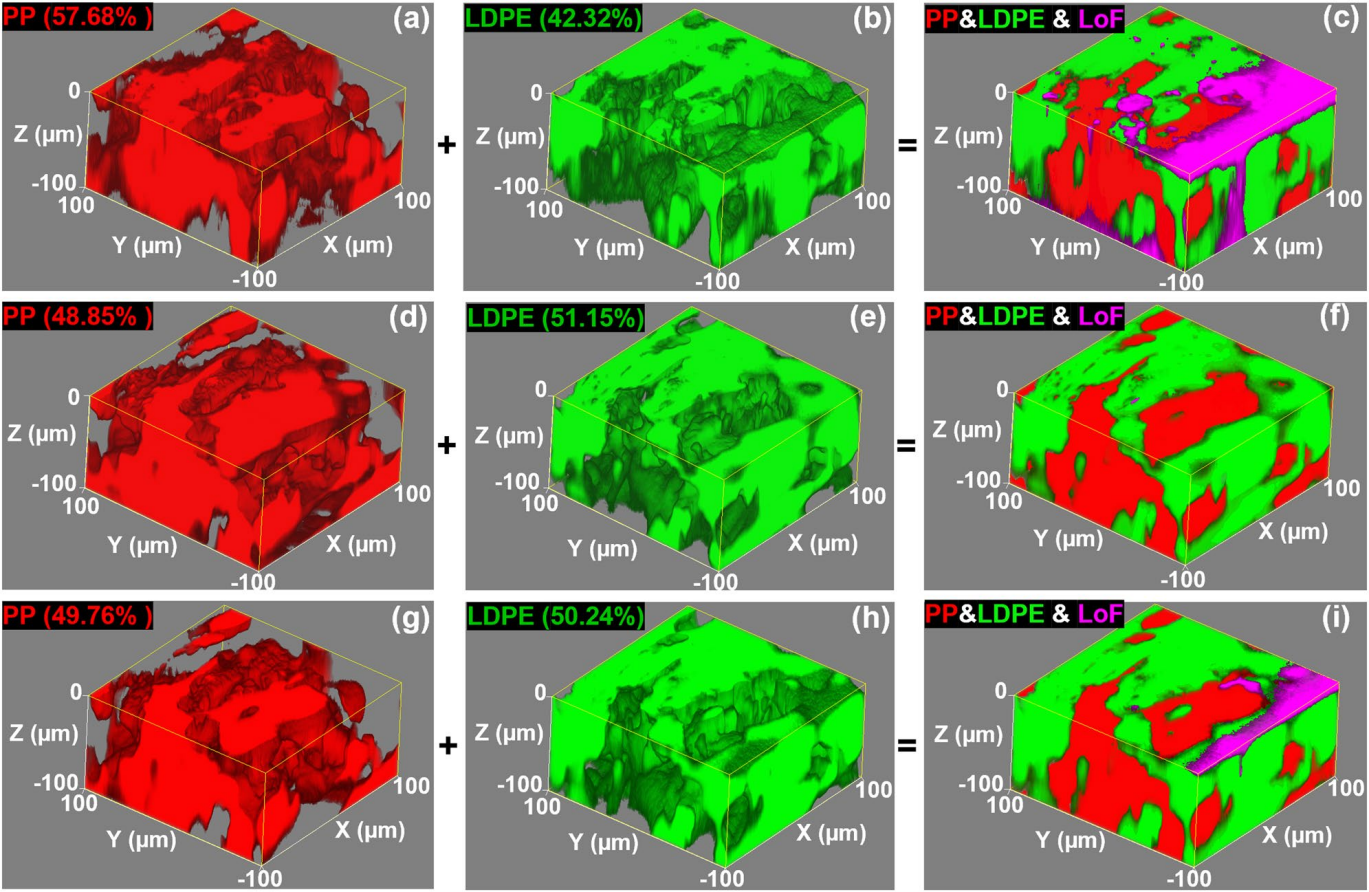
3-D Raman maps of PP/LDPE (50/50) B-MP that are obtained from the same volume using 405 nm (**a**–**c**), 532 nm (**d**–**f**), and 633 nm (**g**–**i**) laser wavelengths. The numbers inside the parenthesis in front of the name of each polymer demonstrate the estimated concentration of that polymer within the mapped volume. LoF stands for “lack of fit” which is shown in purple color.

PP/LDPE (50/50) and PP/LDPE (25/75) B-MPs were targeted to be analyzed with 3-D Raman mapping. It is well known that the lateral and axial resolution of Raman mapping, the penetration depth, the efficiency of both the excitation and collection optics, and the scattering of the Raman signals are all highly dependent on the wavelength of the excitation source^18,30,31^. Therefore, the same volume of the PP/LDPE (50/50) B-MP was 3-D Raman mapped using three different wavelengths including 405, 532, and 633 nm to investigate the effect of the excitation wavelength on the obtained maps, as well as on the precision of the estimated concentration of the constituent polymers. 785 nm excitation wavelength was not used here considering its poorer performance in comparison to the 532 nm wavelength, as shown in the previous section. The results are summarized in Fig. 3. At first glance, more valuable information about the morphology of the distribution of polymers was obtained using this approach which was not feasible using 2-D Raman mapping. Indeed, the morphology was not uniform throughout the different depths of the analyzed B-MP. Comparing the features between the corresponding 3-D maps further confirms that the data were obtained from the same volume during each measurement.

Figure 3c,f,i show the combined image of the complementary Raman maps of each individual component, i.e., PP and LDPE, together with the lack of fit (lof). It was observed that the amount of lof was higher when using the 405 nm excitation wavelength, while it was very low when using the 532 and 633 nm wavelengths. The lower efficiency of the collecting optics for the Raman signal at shorter wavelengths could attribute to it. Nevertheless, lof was masked out from the main data during the quantitative analysis of concentration. Quantitative concentration estimate analysis was applied on the obtained 3-D Raman maps using the reference spectrum of each constituent polymer. The results are shown in the top-left corner of each image. As seen, the precision of the predicted concentrations increased, i.e., became closer to the real amounts, thanks to the more valuable information obtained via 3-D Raman analysis. A similar increase in precision was observed when performing 3-D Raman mapping on PP/LDPE (25/75) B-MP (see Fig. S2). It was also noted that the precision of the quantitative analysis improved by increasing the excitation wavelength. Most likely, that is due to the less scattered Raman signals at longer wavelengths which were collected with higher efficiency. Moreover, the laser spot is typically larger when using a longer wavelength, thus, the Raman signal is collected from a larger volume. Accordingly, the precision of the concentration estimate analysis became higher, as more information was available during the analysis.

**Figure 4:**
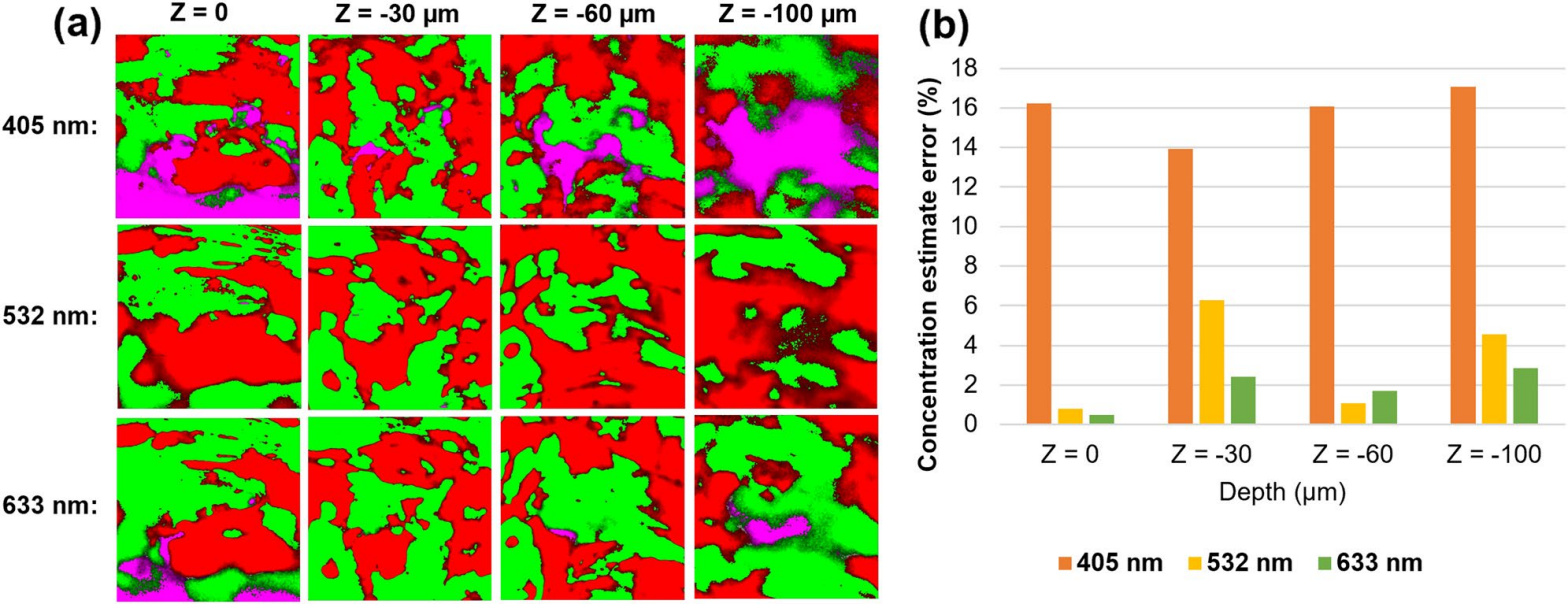
(**a**) shows the 2-D Raman maps exported from different layers of the obtained 3-D Raman maps using 405, 532, and 633 nm wavelengths. (**b**) shows a comparison between the concentration estimate errors (CEE) that are calculated for each corresponding 2-D Raman map shown in (**a**).

To further evaluate these arguments, four 2-D layers starting from the top surface layer to depths of 30, 60, and 100 µm were exported as 2-D Raman maps from the obtained 3-D Raman maps, and subsequently, the corresponding CEE parameter was calculated. The results of this analysis are summarized in Fig. 4. As seen, the CEEs obtained for the 633 nm wavelength were in most cases lower than those obtained for the other two wavelengths, except in Z = 60 µm. The CEEs obtained for the 532 nm wavelength were comparable to the CEEs obtained for the 633 nm, while the CEEs obtained for the 405 nm were the highest in all cases. This again confirms the arguments made above regarding the higher precision of concentration estimate analysis when using the 633 nm wavelength.

Finally, it is important to mention that Raman spectroscopy is well known to be able to discriminate between copolymers with close chemical structures^32,33^. Therefore, the analysis of samples with more complex compositions than what was used in this work is possible using the proposed approach as long as the following conditions are met. First, the reference library that is used to search for the identity of an unknown material is built using high-quality data/spectra of standard materials. Second, the analysis algorithms that are used to interpret the measured data are accurate enough to distinguish subtle differences between similar structures.

### Raman mapping of B-MPs with laser line-focus

**Figure 5:**
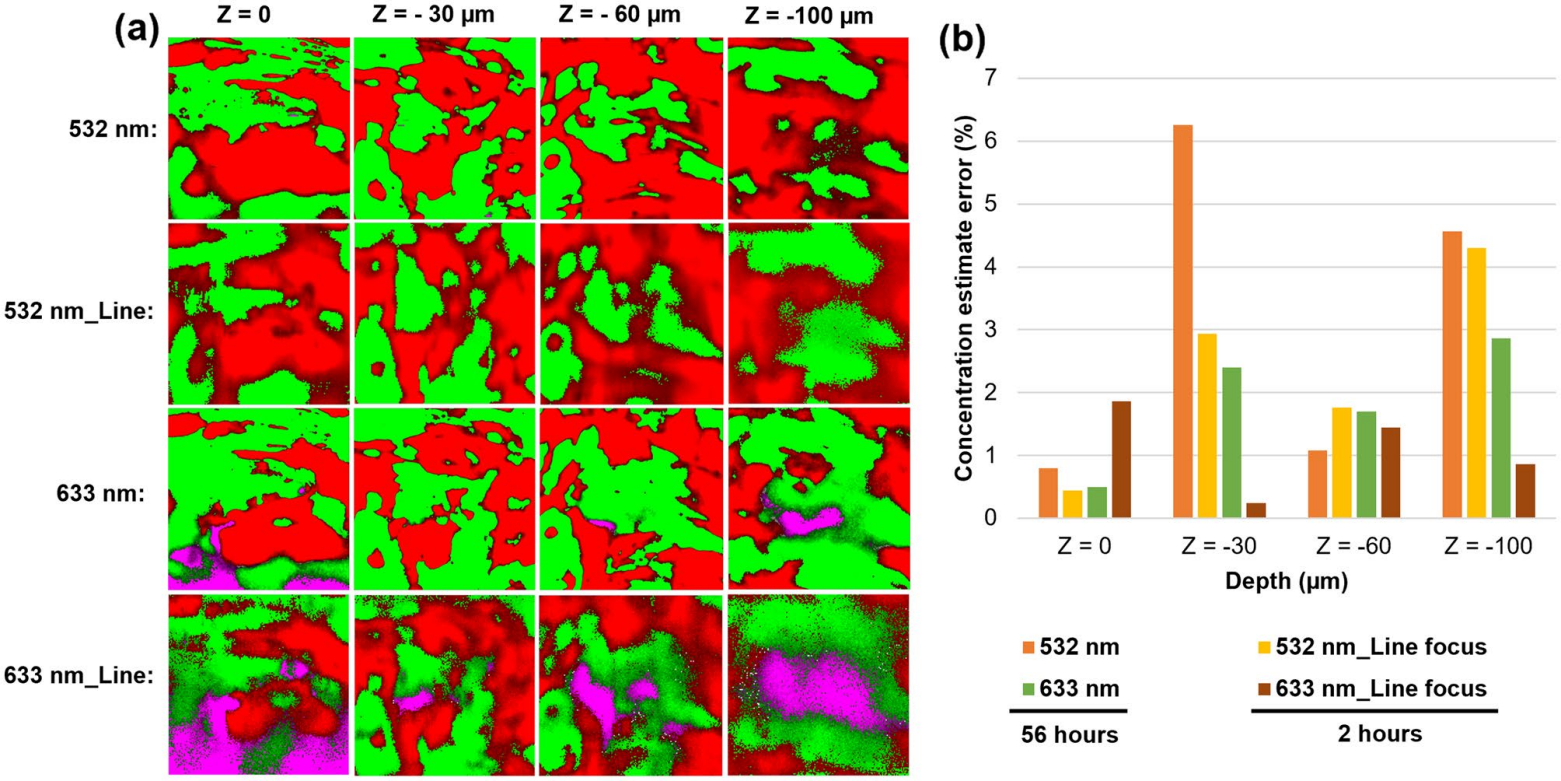
The first and third rows in (**a**) show the 2-D Raman maps exported from different layers of the obtained 3-D Raman maps using 532 and 633 nm wavelengths in the confocal mode, and the second and fourth rows in (**a**) show the obtained 2-D Raman maps of the corresponding layers using 532 and 633 nm laser line-focus. (**b**) Shows a comparison between the concentration estimate errors (CEE) that are calculated for each corresponding 2-D Raman map shown in (**a**).

One of the challenges hampering the application of 3-D Raman mapping for the analysis of MPs or any other sample is the required long measurement time. For example, it takes almost 66 h to obtain the above 3-D Raman maps using each wavelength. Accordingly, we examined the application of laser line-focus to acquire 2-D Raman maps of B-MPs at different depths. The aim was to compromise between the resolution of the obtained Raman maps and the precision of the concentration estimate analysis. Since the CEEs obtained for 405 nm were not as good as for the 532 and 633 nm, the current analysis was only performed with the two latter wavelengths. To make a fair comparison between the results, 2-D Raman maps were obtained using 532 and 633 nm laser line-focus from the same layers of the same sample that were mapped in the confocal mode, i.e., with laser point-focus. Afterward, a concentration estimate analysis was performed on each 2-D Raman map as stated before followed by the calculation of the CEE. The results are summarized in Fig. 5. As expected, the resolution of the mapping using laser line-focus decreased in comparison to the maps obtained in the confocal mode. However, one can still obtain valuable information about the morphology of the distribution of polymers within the different depths of a B-MP using the laser line-focus. For instance, the similarity between features (green and red islands) on the small images on the same columns in Fig. 5a, demonstrates that accurate information about the distribution of polymers can be obtained using Raman mapping with laser line-focus. This is more evident for the 532 nm wavelength due to its higher resolution and especially in the shallower depths. Figure 5b demonstrates the calculated CEEs from the corresponding images shown in Fig. 5a. As seen, the CEEs obtained for the line-focus maps were in most cases either comparable or lower than the CEEs obtained for the point-focus maps. This means that the precision of concentration estimate analysis did not suffer like the resolution of the mapping while using laser line-focus. This again could be due to the collected Raman signal from a larger volume that was excited using the laser line-focus mode.

Finally, utilizing laser line-focus significantly decreased the required time of Raman mapping from 56 to 2 h. Accordingly, one can obtain accurate and fast information about the quantitative concentration of polymers within a B-MP using this approach. It should be noted that faster measurements are possible by sacrificing the signal-to-noise (S/N) ratio of the obtained Raman signal.

## Materials and methods

### Blended microplastic samples

LDPE and PP blended samples were prepared at different mass ratios including 25/75, 50/50, and 75/25, respectively, using simple melt mixing as mentioned elsewhere^15,34^. Briefly, LDPE and PP granules at proper ratios were thoroughly mixed at 170 °C on a steel slide and subsequently cooled down to room temperature for further analysis. The obtained samples were opaque with no color and had an irregular surface shape to mimic the surface properties of real B-MPs (See Fig. S3). The phase separation of the polymers was also observed to be similar to the PP/PE mixed recyclate^28^. The type of all the samples was confirmed using Raman spectroscopy followed by library matching before any analysis.

### Raman setup

Renishaw inVia Basis™ and inVia™ Qontor® Raman microscopes were used throughout this work for the analysis of B-MPs. In total, four different wavelengths including 405, 532, 633, and 785 nm were used to collect the Raman data. 50 × magnification objectives with numerical apertures of either 0.5 (long-working distance) or 0.75 were used where appropriate. The laser power on the sample was adjusted to avoid sample deformation or burning during measurements. 0.5 s was used as the appropriate acquisition time in most of the measurements, providing a good signal-to-noise ratio. Table S1 gives a detailed overview of the measurement settings that were used for each corresponding mapping in this study. Before running any measurement, calibration of the setups was done by measuring the silicon peak (520.5 ± 1) cm^−1^ from a standard reference.

### 2-D and 3-D Raman mapping

2-D Raman mapping was done over a certain area (400 µm × 400 µm) of B-MPs using a raster scan and with a 1 µm step size in X and Y coordinates. The optimum focus was tracked automatically by the instrument during each measurement. Similarly, 3-D Raman mapping was conducted with the same setup and a 10 µm step size in the Z coordinate reaching 100 µm deep inside the B-MPs. An important parameter here was the optical resolution along the path of the laser beam (Z axis) which can be defined using the following formula: $\Delta Z=0.89\lambda /{(N.A.)}^{2}$; where λ is the excitation wavelength and N.A. is the numerical aperture of the microscope objective which was 0.75 in all cases of the performed 3-D Raman mappings. As the system works in the confocal mode, only the light collected from a tiny spot (focal point) is focused onto the photodetector through a small pinhole, which means the light from other depths of the sample is not distorting the signal and is efficiently blocked^31^. Therefore, 10 µm was selected as the step size in the Z direction to avoid oversampling, as well as 100 µm was selected as the final depth to be sure the penetration depth is not exceeded^18^. Figure S4 shows the average signal-to-noise (S/N) ratio of the measured spectra with the shortest wavelength, i.e., 405 nm, at the different depths of B-MPs. As seen, the amount of S/N was still acceptable at a depth of 100 µm. It is important to mention that the use of the autofocusing feature was not employed for the 3-D Raman mapping because of the movement of the stage in the Z coordinate. To fairly compare the results obtained using each excitation wavelength, the same volume of the same B-MP was 3-D mapped during the measurements. Furthermore, a line-shaped laser beam profile (line-focus)^35^ was used to map the same layers of the same B-MP that was mapped in the confocal mode, i.e., with laser point-focus, for subsequent comparison of the results. 2 s was used as an appropriate acquisition time in the latter with 1.3 µm step size in X and Y coordinates.

### Data analysis

All the obtained Raman data were directly processed and analyzed using the embedded modules in WiRE software, the dedicated software for automatic control of Renishaw Raman microscopes. All the cosmic rays were removed from the measured dataset followed by removing the spectral backgrounds using an 11th-order polynomial function. Next, component analysis was done, applying the Non-Negative Least Squares (NNLS) algorithm using the individual spectrum of each polymer that was present in the B-MPs as reference components to plot the 2-D and 3-D Raman maps. The full spectral ranges shown in Table S1 were utilized to analyze the data. It is worth noting that the normalization (mean center and scale to unit variance) of the data was done during this analysis, as well. Renishaw 3-D volume viewer was used to plot the 3-D Raman data. To quantitatively analyze the concentration of the polymers within the B-MPs, concentration estimate analysis was applied on the 2-D and 3-D Raman maps. It is a technique whereby we can calculate the percentage values derived from the least squares fitting of multiple reference spectra, related to the concentration of present species relative to each other. As this analysis was dependent on the intensity of the Raman peaks, the reference spectra were measured with the same parameters as what was used during Raman mapping, and component analysis was performed without normalization of data.

## Conclusion

Considering the large volume market of blended plastics that has developed over a long time, they could form a big portion of the plastics pollution in our environment. Nevertheless, B-MPs have not been the main target of studies so far, which could be due to the lack of a proper analytical method for the study of such complex samples. It was shown here that 3-D Raman microspectroscopy can be a promising technique for the study of B-MPs. It not only provides information about the morphology of the distribution of polymers but also offers the possibility of quantitative analysis of the concentration of each constituent component within B-MPs. Having said that, it is very important to select a proper measurement setting to obtain Raman images with enough resolution and precision for quantitative analysis. To clearly demonstrate this fact, 2-D and 3-D Raman mappings were done with different measurement settings on a relatively large area (400 × 400 µm^2^) and volume (200 × 200 × 100 µm^3^) of B-MPs, respectively. Finally, it was shown that 2-D Raman mapping at different depths of B-MPs with laser line-focus can be an alternative approach to obtain faster information about the distribution of polymers and their concentration.

## Supplementary Information

Supplementary Information.

## Data Availability

The datasets used and/or analyzed during the current study available from the corresponding author on reasonable request.
