# Supplementary material for: Studying the concentration of polymers in blended microplastics using 2D and 3D Raman mapping
Source: Sci Rep. 2023 May 12;13:7771. doi: 10.1038/s41598-023-35010-0 (PMC10182055; doi:10.1038/s41598-023-35010-0)
Supplement: Supplementary file 1 — Supplementary Information. [file 41598_2023_35010_MOESM1_ESM.docx]

**Supplementary Information**

**Studying the concentration of polymers in blended microplastics using 2D and 3D Raman mapping**

Mehrdad Lotfi Choobbari ^a^, Jennifer Ferguson ^b^, Niko Van den Brande ^c^, Tim Smith ^b^, Tatevik Chalyan ^d^, Wendy Meulebroeck ^d^, and Heidi Ottevaere ^d*^

^a^ Vrije Universiteit Brussel, Department of Applied Physics and Photonics, Brussels Photonics, Pleinlaan 2, 1050 Brussels, Belgium

^b^ Renishaw plc, New Mills, Wotton-under-Edge, Gloucestershire, GL12 8JR, United Kingdom

^c^ Vrije Universiteit Brussel, Department of Materials and Chemistry, Physical Chemistry and Polymer Science, Pleinlaan 2, 1050 Brussels, Belgium

^d^ Vrije Universiteit Brussel and Flanders Make, Department of Applied Physics and Photonics, Brussels Photonics, Pleinlaan 2, 1050 Brussels, Belgium

*Corresponding author

Heidi Ottevaere

Phone/fax: +32 472 38 67 12

Email: Heidi.Ottevaere@vub.be

**Table of content**

Table S1. The measurement settings used in this work for acquiring the Raman maps..........S2

Figure S1. 2-D Raman maps of PP/LDPE (25/75) and PP/LDPE (75/25) B-MPs..................S3

Figure S2. 3-D Raman map of PP/LDPE (25/75) B-MP………........……….....…….….....…S4

Figure S3. White-light camera image of the surface of different B-MPs captured with the Renishaw Raman microscope.…………………..................................................................…S4

Figure S4. The average signal-to-noise ratio of the measured Raman spectra using 405 nm excitation wavelength at the different depths of BMPs............................................................S5

Table S1. The measurement settings used in this work for acquiring the Raman maps

| Figure(s) | Laser wavelength (nm) | Average power on sample (mW) | Exposure time (s) | Range (cm^-1^) | Type of focus | Objective | Total time |
| --- | --- | --- | --- | --- | --- | --- | --- |
| 1 & 2 (b-d) | 785 | ~ 30 | 0.5 | 505 – 1631 (1015 points) | Confocal | x50 L | 14 h |
| 1 & 2 (e-g) | 532 | ~ 50 | 0.5 | 165 – 1926 (1021 points) | Confocal | x50 L | 14 h |
| 3 (a-c) | 405 | ~ 25 | 0.5 | 287 – 2550 (1015) | Confocal | x50 | 66 h |
| 3 (d-f) | 532 | ~ 50 | 0.5 | 463 – 1659 (1015) | Confocal | x50 | 66 h |
| 3 (g-i) | 633 | ~ 10 | 0.5 | 484 – 1638 (1015 points) | Confocal | x50 | 66 h |
| 5a (1^st^ row) | 532 | ~ 50 | 0.5 | 463 – 1659 (1015) | Confocal | x50 | 56 h |
| 5a (2^nd^ row) | 532 | ~ 50 | 2 | 463 – 1659 (1015 points) | Line | x50 | 2 h |
| 5a (3^rd^ row) | 633 | ~ 10 | 0.5 | 484 – 1638 (1015 points) | Confocal | x50 | 56 h |
| 5a (4^th^ row) | 633 | ~ 10 | 2 | 484 – 1646 (1023 points) | Line | x50 | 2 h |


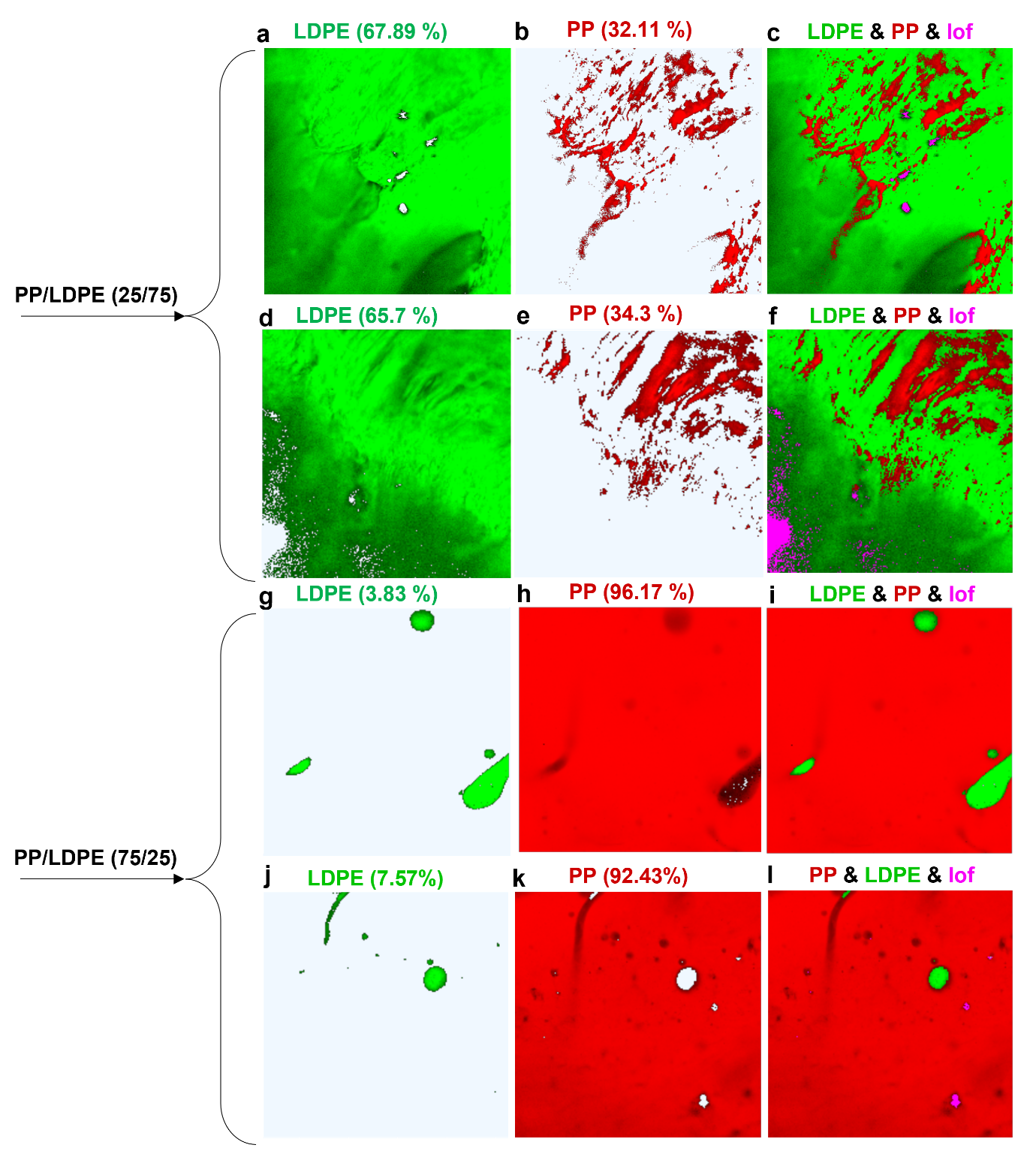


Figure S1. 2-D Raman maps of PP/LDPE (25/75) and PP/LDPE (75/25) obtained with 532 nm excitation wavelength. (a), (d), (g) and (j) show the distribution of LDPE together with its concentration in the corresponding area, (b), (e), (h) and (k) show the distribution of PP together with its concentration in the corresponding area, (c), (f), (i) and (l) show the combined maps of PP and LDPE together with lack of fit (lof) shown in purple color. Mapping area is 400 µm × 400 µm.


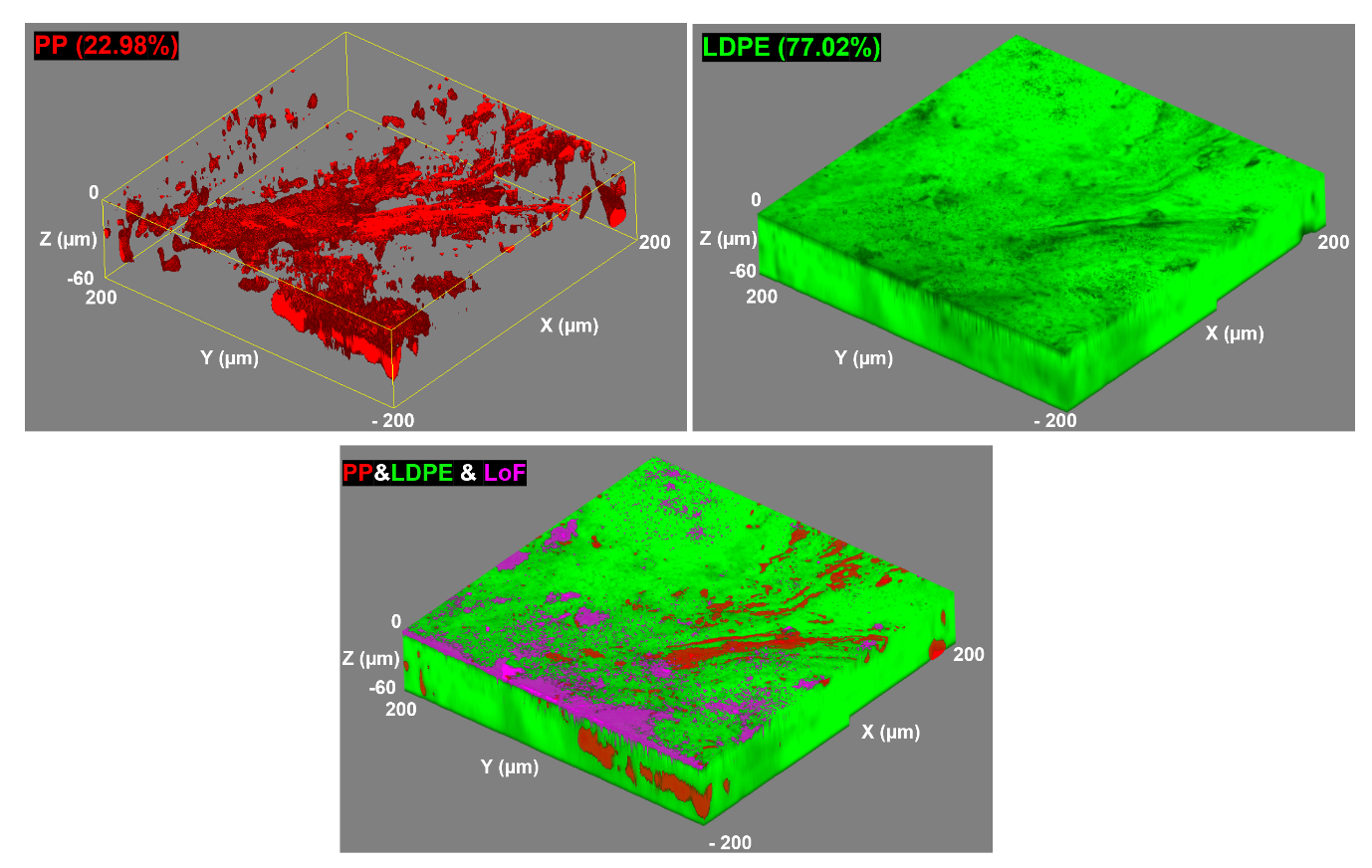


Figure S2. 3-D Raman map of PP/LDPE (25/75) B-MP that is obtained using 532 nm excitation wavelength. The numbers inside the parenthesis in front of the name of each polymer demonstrate the estimated concentration of that polymer within the mapped volume. LoF stands for “lack of fit” which is shown in purple color.


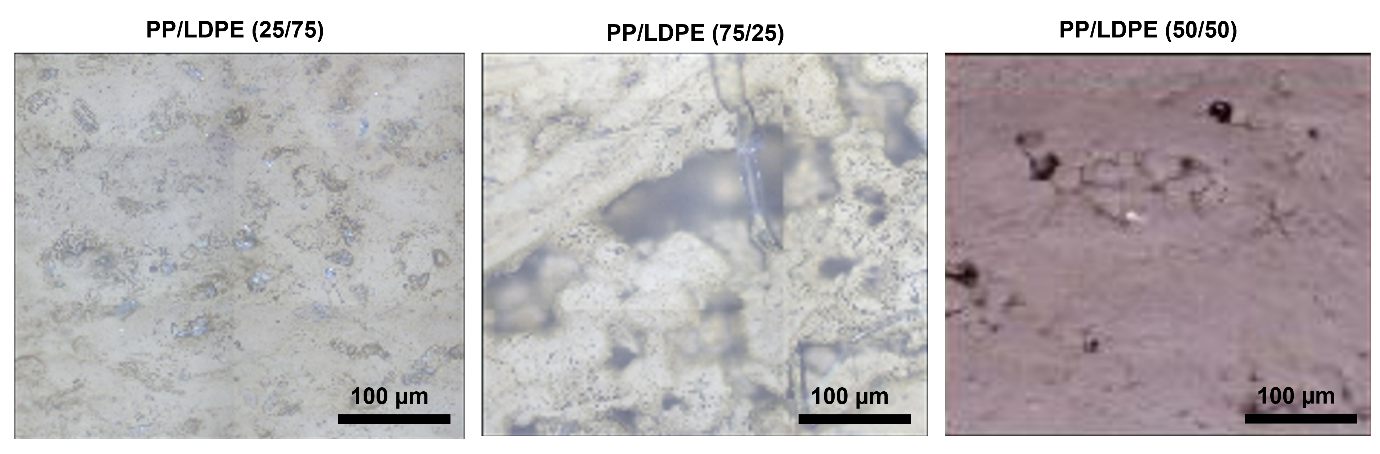


Figure S3. White-light camera image of the surface of different B-MPs captured with the Renishaw Raman microscope.


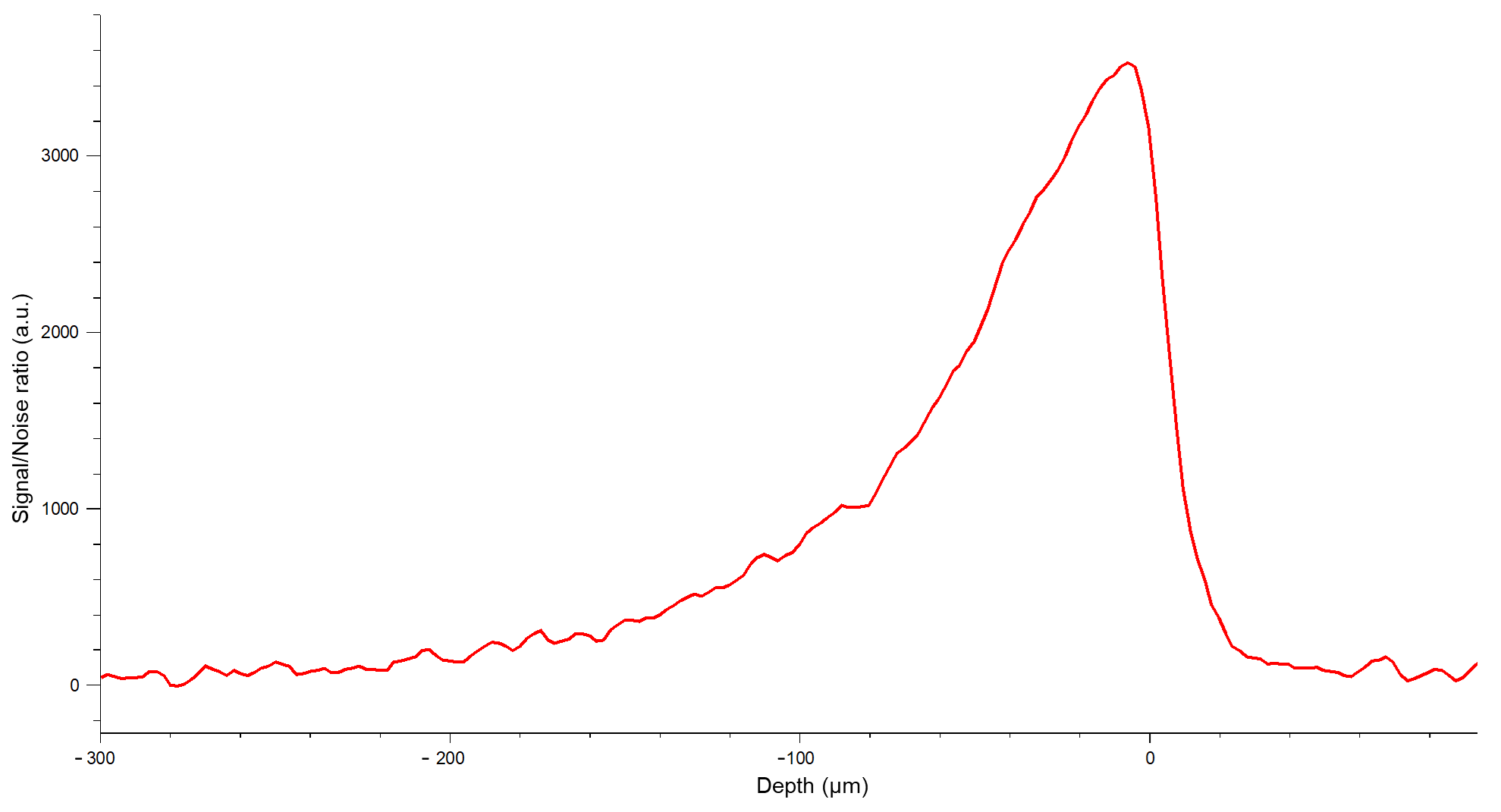


Figure S4. The average signal-to-noise ratio of the measured Raman spectra using 405 nm excitation wavelength at the different depths of BMPs.
